# Central and effector memory T cells in peripheral blood of patients with interstitial pneumonia: preliminary clues from a COVID-19 study

**DOI:** 10.1186/s12931-022-02190-8

**Published:** 2022-10-10

**Authors:** Makhabbat Bekbossynova, Lyudmila Akhmaltdinova, Kuanysh Dossybayeva, Ainur Tauekelova, Zauresh Smagulova, Tatyana Tsechoeva, Gulsimzhan Turebayeva, Aliya Sailybayeva, Zhanar Kalila, Tahmina Mirashirova, Timur Muratov, Dimitri Poddighe

**Affiliations:** 1 National Research Cardiac Surgery Center, 010000 Nur-Sultan, Kazakhstan; 2 Nazarbayev University School of Medicine (NUSOM), Kerei-Zhanibek Str. 5/1, 010000 Nur-Sultan, Kazakhstan; 3 City Infectious Disease Center at Multidisciplinary Medical Center, 010000 Nur-Sultan, Kazakhstan; 4 Department of Public Health of Nur‑Sultan City, 010000 Nur-Sultan, Kazakhstan; 5 University Medical Center (UMC), 010000 Nur-Sultan, Kazakhstan

**Keywords:** Memory T cells, Interstitial pneumonia, COVID-19, SARS-CoV-2, Central memory T cells, Effector memory T cells, Lymphocyte subpopulations

## Abstract

**Background:**

SARS-CoV-2 pre-existing T-cell immune reactivity can be present in some people. A general perturbation of the main peripheral lymphocyte subsets has been described in severe COVID-19 patients, but very few studies assessed the general memory T-cell homeostasis in the acute phase of COVID-19. Here, we performed a general analysis of the main memory T cell populations in the peripheral blood of patients admitted to the hospital for a confirmed or probable COVID-19 diagnosis.

**Methods:**

In this cross-sectional study, adult patients (aged ≥ 18 years) needing hospital admission for respiratory disease due to confirmed or probable COVID-19, were recruited before starting the therapeutic protocol for this disease. In addition to the assessment of the general lymphocyte subpopulations in the early phase of COVID-19, central memory T cells (Tm_centr_ cells: CD45RO+CCR7+) and effector memory T cells (Tm_eff_ cells: CD45RO+CCR7−) were assessed by multi-color flow cytometry, in comparison to a control group.

**Results:**

During the study period, 148 study participants were recruited. Among them, 58 patients turned out positive for SARS-CoV-2 PCR (including both patients with interstitial pneumonia [PCR+Pn+] and without this complication [PCR+Pn−]), whereas the remaining 90 patients resulted to be SARS-CoV-2 PCR negative, even though all were affected with interstitial pneumonia [PCR−Pn+]. Additionally, 28 control patients without any ongoing respiratory disease were recruited. A clear unbalance in the T memory compartment emerged from this analysis on the whole pool of T cells (CD3+ cells), showing a significant increase in Tm_centr_ cells and, conversely, a significant decrease in Tm_eff_ cells in both pneumonia groups (PCR+Pn+ and PCR−Pn+) compared to the controls; PCR+Pn− group showed trends comprised between patients with pneumonia (from one side) and the control group (from the other side). This perturbation inside the memory T cell compartment was also observed in the individual analysis of the four main T cell subpopulations, based upon the differential expression of CD4 and/or CD8 markers.

**Conclusion:**

Overall, we observed both absolute and relative increases of Tm_centr_ cells and decrease of Tm_eff_ cells in patients affected with interstitial pneumonia (regardless of the positive or negative results of SARS-CoV-2 PCR), compared to controls. These results need confirmation from additional research, in order to consider this finding as a potential biological marker of interstitial lung involvement in patients affected with viral respiratory infections.

## Introduction

Since the first description of SARS-CoV-2 in December 2019 and the declaration of COVID-19 pandemic in March 2020, > 500 million confirmed cases and > 6 million related deaths have been registered all over the world [1].

The clinical impact of SARS-CoV-2 infection can be extremely variable, ranging from an asymptomatic condition (or mild upper respiratory symptoms) to a rapidly progressive clinical course characterized by the development of interstitial pneumonia, which can lead to respiratory failure and acute respiratory distress syndrome (ARDS) [2, 3].

In addition to COVID-19, acute interstitial pneumonia can be associated with several respiratory viruses (including influenza, parainfluenza, adenovirus, and others) and some bacterial infections (such as *Mycoplasma pneumoniae*, *Chlamydophila pneumoniae*, *Legionella pneumophila*) [3]. However, SARS-CoV-2 more frequently (than other etiologic agents) leads to severe clinical forms in patients with risk factors, such as older age, obesity, cardiovascular diseases, diabetes, immunosuppression, and others [3, 4].

Multiple innate and adaptive components of the immune system are involved in the immunological response to SARS-CoV-2. Although the rapid clinical course seen in COVID-19 could indicate that the infection control in asymptomatic or mildly affected patients may be due to an effective anti-viral innate immune response, the role of the adaptive immune system and, in detail, T-cell compartment is also relevant from the first phases of SARS-CoV-2 infection [5, 6]. Indeed, a general perturbation of the main peripheral lymphocyte subsets has been described in severe COVID-19 patients, which may further impact on and/or be associated with the clinical expression of SARS-CoV-2 infection in different patients [6, 7]. There is evidence that SARS-CoV-2 pre-existing T-cell immune reactivity can be present to some degree in some people (probably due to previous exposure to other  coronaviruses), and several studies investigated the existence and dynamics of antigen-specific T cells against SARS-CoV-2 (especially in convalescent COVID-19 and/or long-COVID-19 patients) [8, 9]. However, very few studies assessed the general memory T-cell homeostasis in the acute phase of COVID-19.

In this preliminary study, we performed a general analysis of the main memory T cell populations (in detail, central and effector T memory cells) in the peripheral blood of patients admitted to the hospital due to a confirmed or probable COVID-19 diagnosis.

## Materials and methods

### Study design and patients

In this cross-sectional study, adult patients (aged ≥ 18 years) needing medical care and hospital admission for respiratory disease due to confirmed or probable COVID-19, were recruited. These patients were admitted to the Department of Infectious Diseases (Sub-intensive and Intensive Care Units) of the City Infectious Diseases Center (CIDC) of Nur-Sultan (Kazakhstan) in the study period from February 14th until June 15th, 2022.

Additionally, adult patients admitted to the Cardiology, Arrhythmic Disorders and Cardiac Surgery Departments at the National Research Center for Cardiac Surgery (NRCCS), Nur-Sultan (Kazakhstan) were recruited as a control group: all these patients were hospitalized for a routine cardiological assessment; they were negative for RT-SARS-CoV-2 PCR and did not have any acute and ongoing respiratory disease.

All these patients gave consent to donate a small amount of blood (3–4 mL) for additional flow cytometry analysis of lymphocytes, in order to be recruited for this research. All patients received SARS-CoV-2 RT-PCR test before or at the hospital admission. Even though these patients underwent SARS-CoV-2 RT-PCR test (henceforth abbreviated as PCR) in different laboratories, all tests were performed with certified diagnostic kits and authorized laboratories according to the rules approved by the Ministry of Health of Republic of Kazakhstan (№ KR DSM-114 from November 12th, 2021).

The following exclusion criteria were established: lack of informed consent to participate in this study; anamnestic and/or clinical evidence of pre-existing interstitial lung disease; presence of specific comorbidities (oncological disorders, hematological disorders, HIV infection, chronic renal failure with dialysis, any disease previously treated with biological or immunosuppressive therapy; previous treatment with intravenous immunoglobulin or plasma derivatives); pregnancy.

Confirmed COVID-19 patients were diagnosed according to the World Health Organization COVID-19 case definitions and, accordingly, all these patients resulted to be SARS-CoV-2 PCR positive. Patients with respiratory disease and negative SARS-CoV-2 PCR were admitted to the hospital as suspected or probable COVID-19 cases. All these patients with respiratory disease were classified in terms of COVID-19 severity according to the WHO criteria [10, 11].

### Ethical statement

The study was conducted according to the guidelines of the Declaration of Helsinki and approved by local Ethics Committee of the NRCCS (Protocol no. 01-91/2021 dated on April 22nd, 2021) in agreement with the ethical principles of the State Standard for Good Clinical Practice, Rules for conducting biomedical research and requirements for research centers from December 21, 2020, № KR DSM-310/2020. Written and signed informed consent was obtained of all patients involved in the study (or legally authorized guardian, if appropriate).

### Clinical, laboratory and radiological data

Upon admission to the hospital, these patients underwent a complete clinical examination (including an accurate collection of personal and family history) and first-level diagnostic work-up (including a complete blood cell count—CBC, erythrocyte sedimentation rate—ESR, serum C-reactive protein—CRP).

All patients with respiratory disease received a chest computerized tomography (CT) to assess presence, type, and extension of lung involvement. Chest CT was performed by Ingenuity CT, Philips Healthcare, Cleveland, USA. Control patients were negative for respiratory symptoms and, therefore, did not undergo chest CT; however, because of their cardiological check-up, all these patients received a chest X-ray, which ruled out ongoing bronchitis and pneumonia.

In addition to PCR test, all study participants received a specific SARS-CoV-2 serological assessment (by the paramagnetic particle chemiluminescent immunoassay, CLIA) in terms of anti-SARS-CoV-2 IgM (iFlash-SARS-CoV-2 IgM, Shenzhen YHLO Biotech Co. Ltd., cut-off 10 AU/mL), anti-SARS-CoV-2 IgG (iFlash-SARS-CoV-2 IgG-S, Shenzhen YHLO Biotech Co. Ltd., cut-off 10 AU/mL, measuring range 2.00–3500 AU/mL), and anti-S-RBD SARS-CoV-2 Neutralization Antibody (NAb) (iFlash-2019-nCoV NAb, Shenzhen YHLO Biotech Co. Ltd., cut-off 10 AU/mL, measuring range 4.00–800 AU/mL).

Finally, all study participants underwent flow cytometric analysis to assess the general lymphocyte populations and, in detail, the T-memory compartment, as described in the following subsection.

### Flow cytometry analysis

Lymphocyte subpopulations analysis by flow cytometry was performed on the same day of blood collection. Peripheral blood mononuclear cells (PBMCs) were isolated from EDTA whole blood (3–4 mL) by using Ficoll-Paque PLUS (Cytiva) density gradient centrifugation. Remaining erythrocytes were lysed with ACK lysing buffer (Gibco TM, ThermoFisher). Isolated PBMC were labelled with the following conjugated monoclonal antibodies: CD3-AF750 (UCHT1), CD4-APC (13B8.2), CD8-AF750 (B9.11), CD45-KrO (J33), CD45RO-FITC, (UCHL1), CCR7/CD197-PC7 (G043H7); and: CD3-AF750 (UCHT1), CD4-APC (13B8.2), CD8-FITC (B9.11), CD19-PE (J3-119), CD56-PC5.5 (N901), CD45-KrO (J33) (Immunotech SAS, Beckman Coulter, Marseille, France). Then, cells were resuspended and examined using a DX Flex flow cytometer (Beckman Coulter, USA). The resulting data was analyzed using CytoFlex, Kaluza 2.1 (Beckman Coulter). Forward and side scatter was used to distinguish the lymphocyte population in addition to the signal from CD45. The distribution of markers CD3, CD4 and CD8 was analyzed in the pool of T-lymphocytes. To analyze the status of memory T cells, CCR7/CD197 and CD45RO markers were used.

### Statistical analysis

Data collection and descriptive analysis were carried out by Microsoft^®^ Excel 2010 for Windows. Inter-groups differences in categorical variables were statistically analyzed through Chi-square test; p-value < 0.05 was considered statistically significant. Continuous variables were expressed as mean (M) ± standard deviation (SD). Differences between two groups were analyzed by two-tailed unpaired t-test (with Welch’s correction). The analysis among 3 groups or more was performed by ordinary one-way ANOVA, if no heterogeneity of variances was confirmed (based upon Brown–Forsythe test); the multiple comparison of the mean of each group with the mean of every other group used the Tukey post-test. The analysis among 3 groups or more was performed by Welch’s one-way ANOVA, if heterogeneity of variances was observed (based upon Brown–Forsythe test); the multiple comparison of the mean of each group with the mean of every other group used the Dunnett post-test. A p-value < 0.05 was considered as statistically significant. Overall ANOVA test p-values are reported in the tables, whereas relevant post-test p-values (related to the comparisons between two specific groups) are expressed in the text and/or graphically summarized in the figures (if statistically significant). The statistical analysis and elaboration was made by Prism 9 for MacOS (version 9.3.1, GraphPad Software).

## Results

### Demographics characteristics

During the study period, 148 patients who were hospitalized due to acute respiratory disease, accepted to participate in this research.

At the hospital admission, all these 148 patients underwent SARS-CoV-2 PCR test. Among them, 58 patients turned out positive for SARS-CoV-2 PCR and were admitted as confirmed cases of COVID-19 (SARS-CoV-2 PCR+ respiratory disease group). According to the WHO guidelines for COVID-19 disease severity, these patients were classified as affected by mild (n = 19, 32.8%), moderate (n = 33, 56.9%), and severe (n = 6, 10.3%) disease. Conversely, all other 90 patients resulted to be SARS-CoV-2 PCR negative and were admitted as cases of probable COVID-19, due to the evidence of interstitial pneumonia and epidemiological criteria (SARS-CoV-2 PCR− respiratory disease group). Additionally, 28 control patients without any ongoing respiratory disease (and, of course, SARS-CoV-2 PCR negative) and any evidence of other concomitant infections, were recruited among patients who were hospitalized for a regular cardiological check-up and accepted to participate in this research (control group).

The main demographic characteristics of these three initial groups (based upon the recruitment criteria and PCR results) are summarized in Table 1. These three groups are not significantly different in gender, but they show a statistically significant difference in terms of age (p = 0.0203): in detail, SARS-CoV-2 PCR− and PCR+ respiratory disease groups resulted to be mildly different for age (p = 0.0172). By definition, the control group differs (p = 0.0057) from both respiratory disease groups in terms of cardiological comorbidities (except for arterial hypertension); however, the rate and type of cardiological problems between SARS-CoV-2 PCR− and PCR+ respiratory disease groups were similar. Chronic metabolic, respiratory, and renal disorders were similarly represented in all these three groups. Notably, no significant differences were observed among these three groups in terms of SARS-CoV-2 immunization rate.

**Table 1:** Main demographic, clinical and serological characteristics of the study population

	SARS-CoV-2 PCR+ Respiratory Disease Group	SARS-CoV-2 PCR− Respiratory Disease Group	Controls	p-value
Patients [n (%)]	58	90	28	–
Pneumonia cases [n (%)]	39 (67.2)	90 (100)	0 (0)	< 0.001
SARS-CoV-2 IgM+ cases [n (%)]	10 (17.2)	0 (0)	0 (0)	< 0.001
SARS-CoV-2 IgG+ cases [n (%)]	49 (84.5)	86 (95.6)	25 (89.3)	ns
SARS-CoV-2 IgG RBD+ cases [n (%)]	35 (60.3)	84 (93.3)	25 (89.3)	ns
SARS-CoV-2 IgM [AU/mL]	9.55 ± 22.61	0.84 ± 1.38	1.84 ± 2,60	0.0043
SARS-CoV-2 IgG [AU/mL]	688.2 ± 954.0	1099.1 ± 990.8	1149.0 ± 1162.0	0.0342
SARS-CoV-2 IgG RBD [AU/mL]	334.2 ± 383,0	437.1 ± 447.1	364.3 ± 369.8	ns
Age [years]	57.2 ± 14.1	49.8 ± 16.3	50.9 ± 18.3	0.0206
Gender—F/M [n]	39/19	57/33	16/12	ns
SARS-CoV-2 vaccinated pts. [n (%)]	29 (50.0)	53 (58.9)	11 (39.3)	ns
Chronic comorbidities				
Cardiovascular diseases [n (%)]	31 (53.4)	43 (47.8)	28 (100)	0.0057
Primary cardiomyopathy	3 (5.2)	2 (2.2)	9 (32.1)	0.0001
Ischemic cardiomyopathy	7 (12.1)	8 (8.9)	12 (42.8)	0.0002
Hypertension	29 (50.0)	40 (44.4)	19 (67.8)	ns
Cardiac arrhythmias	4 (6.9)	4 (4.4)	9 (32.1)	0.0002
Congenital heart disease	0 (0%)	0 (0)	6 (21.4)	0.0001
Metabolic diseases [n (%)]	25 (43.1)	34 (37.8)	8 (28.6)	ns
Diabetes mellitus type 2	7 (12.1)	613 (14.4)	2 (6.6)	ns
Obesity	19 (32.8)	29 (32.2)	8 (28.6)	ns
Respiratory diseases [n (%)]	3 (7.6)	8 (8.9)	1 (3.6)	ns
Asthma	1 (1.7)	6 (6.7)	1 (3.6)	ns
COPD	2 (3.4)	2 (2.2)	(0)	ns
Renal diseases [n (%)]	3 (5.2)	5 (5.6)	4 (13.3)	ns

### SARS-CoV-2 specific serology

As a part of this study, all participants received the evaluation of SARS-CoV-2 specific serology, as described in Table 1. In the SARS-CoV-2 PCR+ respiratory disease group, only 10 (17.2%) patients concomitantly showed an increased titer of SARS-CoV-2 specific IgM (9 of them were diagnosed with pneumonia), whereas all patients of both other groups resulted IgM negative. In general, the serum levels of specific SARS-CoV-2 IgM in patients of the SARS-CoV-2 PCR+ respiratory disease group (9.55 ± 22.61 AU/mL) was much greater than that observed in the other two SARS-CoV-2 PCR− groups (SARS-CoV-2 PCR+ respiratory disease group: 0.84 ± 1.38 AU/mL, p = 0.0145; control group: 1.84 ± 2.60 AU/mL, p = 0.0382). Notably, despite a similar immunization rate, a mild difference (p = 0.0438) in the SARS-CoV-2 specific IgG titer was observed between SARS-CoV-2 PCR- and PCR+ respiratory disease groups. No inter-groups differences were observed in terms of SARS-CoV-2 IgG RBD.

In summary, the serological pattern and, in detail, the IgM titer specific to SARS-CoV-2 was concordant with SARS-CoV-2 PCR results: only some patients with positive SARS-CoV-2 PCR also resulted SARS-CoV-2 IgM+, whereas all SARS-CoV-2 PCR− patients were also SARS-CoV-2 IgM-. Therefore, all together these findings suggested that the final diagnosis of interstitial pneumonia in SARS-CoV-2 PCR− patients could not be COVID-19, unlike the diagnosis received at the hospital admission.

### Lung involvement (interstitial pneumonia)

As explained above, all 58 patients included in the SARS-CoV-2 PCR+ respiratory disease group were admitted with a diagnosis of confirmed COVID-19 and were assessed by lung CT, which revealed interstitial pneumonia in 39 cases (67.2%). These SARS-CoV-2 PCR+ patients affected with pneumonia (PCR+Pn+) showed a lung damage ranging from 4% to 75%, according to the radiological reports. Conversely, the remaining 19 SARS-CoV-2 PCR+ patients showed no lung involvement (PCR+Pn−), but were admitted for monitoring the respiratory disease, due to the ascertained diagnosis of COVID-19. All the 90 patients included in the SARS-CoV-2 PCR− respiratory disease group were admitted as probable cases of COVID-19, based on epidemiological criteria and evidence of interstitial pneumonia in all of them (which was the main reason for the hospital admission). Therefore, this group can be indicated as PCR−Pn+: these patients showed a lung damage ranging from 4% to 60%. All 30 control patients had no respiratory disease by definition (controls).

**Fig. 1:**
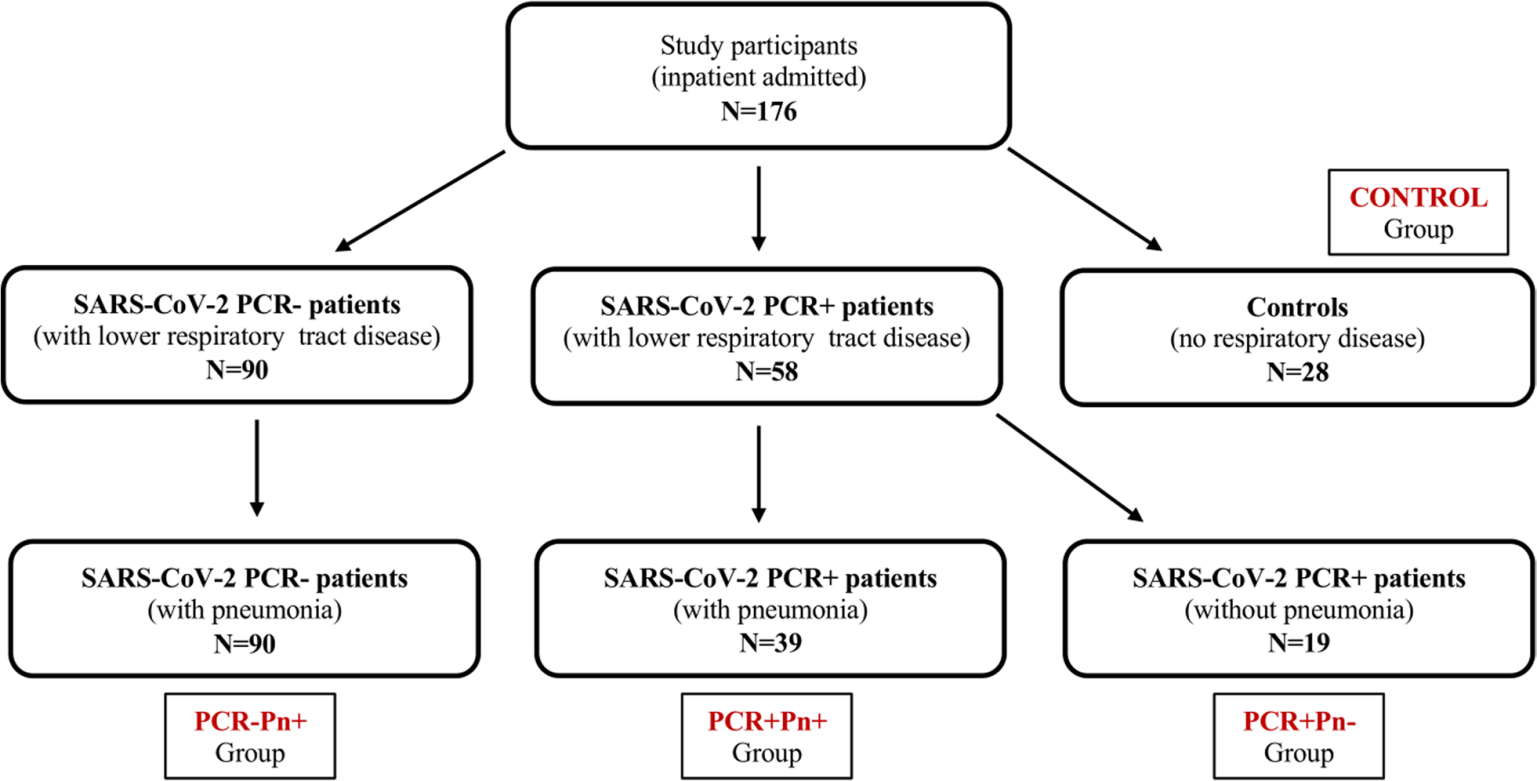
Study groups based on SARS-CoV-2 PCR and radiological results

Therefore, based on the combination of the SARS-Cov-2 PCR test results and the radiological assessment, our study population can be more appropriately considered as composed by 4 main study groups (namely PCR+Pn+, PCR+Pn−, PCR−Pn−, controls), as graphically summarized in Fig. 1.

The comparison of the main demographic, clinical, and serological characteristics between the two specific SARS-CoV-2 PCR+ groups (PCR+Pn+ and PCR+Pn−) is shown in Table 2. PCR+Pn+ patients were significantly older (p = 0.0037) than PCR+Pn− patients, but no difference in gender ratio was observed. Moreover, cardiovascular comorbidity was more frequent (p = 0.0483) in PCR+Pn+ patients, but this association is mainly explained by the higher prevalence of arterial hypertension (p = 0.0075), whereas all other cardiovascular comorbidities were not significantly different between these two groups. As regards the SARS-CoV-2 serological profile, SARS-Cov-2 specific IgM levels were mildly increased in PCR+Pn+ patients (p = 0.0213), who conversely showed remarkably lower levels of SARS-CoV-2 specific IgG RBD compared to PCR+Pn− group, although the total levels of SARS-CoV-2 specific IgG were not significantly different, as well as the immunization rate.

**Table 2:** Main demographic, clinical and serological characteristics of PCR+ groups

SARS-CoV-2 PCR+ groups			
	PCR+Pn+ group	PCR+Pn− group	p-value
Patients [n (%)]	39	19	–
SARS-CoV-2 IgM+ cases [n (%)]	7 (17.9)	3 (15.8)	ns
SARS-CoV-2 IgG+ cases [n (%)]	33 (84.6)	16 (84.2)	ns
SARS-CoV-2 IgG RBD+ cases [n (%)]	25 (64.1)	10 (52.6)	ns
SARS-CoV-2 IgM [AU/mL]	12.99 ± 26.93	2.50 ± 3.26	0.0213
SARS-CoV-2 IgG [AU/mL]	543.2 ± 913,7	985.7 ± 990.3	ns
SARS-CoV-2 IgG RBD [AU/mL]	203.6 ± 344.1	617.1 ± 307.7	< 0.0001
Age [years]	60.9 ± 11.9	49.7 ± 15.8	0.0037
Gender—F/M [n]	28:11	11:8	ns
SARS-CoV-2 vaccinated pts. [n (%)]	17 (43.5%)	12 (63%)	ns
Chronic comorbidities			
Cardiovascular diseases [n (%)]	26 (66.7)	5 (26.3)	0.0483
Primary cardiomyopathy	3 (7.7)	0 (0)	ns
Ischemic cardiomyopathy	7 (17.9)	0 (0)	ns
Hypertension	24 (61.5)	5 (26.3)	0.0075
Cardiac arrhythmias	4 (10.3)	0 (0)	ns
Congenital heart disease	0 (0)	0 (0)	ns
Metabolic diseases [n (%)]	19 (48.7)	6 (31.6)	ns
Diabetes mellitus type 2	6 (15.4)	1 (5.3)	ns
Obesity	14 (3.9)	5 (26.3)	ns
Respiratory diseases [n (%)]	2 (5.1)	1 (5.3)	ns
Asthma	1 (2.6)	0 (0)	ns
COPD	1 (2.6)	1 (5.3)	ns
Renal diseases [n (%)]	2 (5.1)	1 (5.3)	ns

### Leukocytes and acute inflammatory parameters

The main hematological and inflammatory parameters of these final 4 study groups are described in Table 3. In general, white blood cells (WBC) and neutrophils (NEUT) were more increased in PCR-Pn+ patients compared to both PCR+Pn+ and PCR+Pn− groups and, indeed, only the former group showed a statistically significant difference compared to controls (WBC: 9.04 ± 3.30 * 10^9^/L vs. 6.34 ± 1.61 * 10^9^/L respectively, p = 0.0003; NEUT: 6.03 ± 3.09 * 10^9^/L vs. 3.91 ± 1.29 * 10^9^/L, respectively, p < 0.0001). However, in relative terms (expressed as leukocytes percentage), the neutrophil proportion was similar in both pneumonia (PCR−Pn+ and PCR+Pn+) groups; moreover, the PCR+Pn+ patients showed a greater percentage of neutrophils than PCR+Pn− patients (respectively, 67.38 ± 11.51% vs. 56.38 ± 12.30%, p = 0.0147).

**Table 3:** Main hematological (including general lymphocyte populations) and acute inflammatory parameters in the study groups

	PCR−Pn+	PCR+Pn+	PCR+Pn−	Controls	p-value
Patients (n)	90	39	19	28	
WBC (10^9^/L)	9.04 ± 3.30	7.54 ± 3.28	7.32 ± 2.55	6.34 ± 1.61	< 0.0002
NEUT (10^9^/L)	6.03 ± 3.09	5.24 ± 2.98	4.13 ± 1.93	3.91 ± 1.29	< 0.0001
*NEUT (%)*	64.88 ± 17.70	67.38 ± 11.51	56.38 ± 12.30	61.13 ± 7.84	0.0059
LYMPH (10^9^/L)	1.99 ± 0.96	1.60 ± 0,62	2.22 ± 0.75	1.75 ± 0.54	0.0066
*LYMPH (%)*	*24.12* ± *12.12*	*22.74* ± *8.47*	*33.06* ± *10.50*	*27.96* ± *6.58*	*0.0010*
MONO (10^9^/L)	0.84 ± 0.42	0.65 ± 0,32	0.56 ± 0.21	0.51 ± 0.15	0.0001
*MONO (%)*	*9.86* ± *4.59*	*9.17* ± *4.43*	*8.14* ± *2.21*	*8.24* ± *1.66*	*0.0318*
ESR (mm/h)	22.7 ± 12.6	25.7 ± 11.9	10.6 ± 9.8	–	0.0035
CRP (mg/dL)	2.73 ± 4.45	2.60 ± 3.12	0.48 ± 1.20	0.51 ± 0.15	0.0001
*T cells (%)*	*69.07* ± *11.93*	*69.07* ± *11.24*	*67.41* ± *10.28*	*72.46* ± *9.28*	*ns*
T cells (10^9^/L)	1.371 ± 0.687	1.107 ± 0.478	1.493 ± 0.530	1.255 ± 0.397	ns
*T CD4+ cells (%)*	*49.70* ± *12.90*	*49.57* ± *12.14*	*45.86* ± *12.18*	*52.67* ± *9.04*	*ns*
T CD4+ cells (10^9^/L)	0.994 ± 0.558	0.803 ± 0.401	1.014 ± 0.444	0.894 ± 0.233	ns
*T CD8+ cells (%)*	*15.39* ± *7.59*	*14.93* ± *8.20*	*16.32* ± *9.17*	*15.29* ± *6.70*	*ns*
T CD8+ cells (10^9^/L)	0.300 ± 0.206	0.229 ± 0.138	0.365 ± 0.225	0.282 ± 0.197	ns
*DNT cells (%)*	*2.30* ± *1.80*	*2.67* ± *1.75*	*3.54* ± *2.11*	*2.54* ± *0.89*	*0.0388*
DNT cells (10^9^/L)	0.045 ± 0.050	0.044 ± 0.039	0.079 ± 0.049	0.046 ± 0.025	0.0262
*DPT cells (%)*	*1.02* ± *0.86*	*1.16* ± *0.91*	*0.90* ± *0.58*	*1.07* ± *1.15*	*ns*
DPT cells (10^9^/L)	0.020 ± 0.018	0.020 ± 0.020	0.019 ± 0.011	0.017 ± 0.014	ns
*NK cells (%)*	*11.42* ± *8.81*	*14.42* ± *8.32*	*17.00* ± *8.75*	*14.92* ± *9.24*	*0.0308*
NK cells (10^9^/L)	0.218 ± 0.184	0.217 ± 0.130	0.385 ± 0.271	0.273 ± 0.209	0.0040
*B cells (%)*	*15.86* ± *9.02*	*13.08* ± *7.89*	*13.49* ± *7.52*	*10.42* ± *4.39*	*0.0140*
B cells (10^9^/L)	0.325 ± 0.259	0.209 ± 0.150	0.291 ± 0.176	0.183 ± 0.090	0.0003

Overall, both pneumonia groups (PCR−Pn+ and PCR+Pn+) showed lower absolute and, to a greater extent, relative lymphocyte (LYMPH) count compared to the PCR+Pn− group. Notably, among SARS-CoV-2 PCR positive patients, PCR+Pn+ group showed significantly lower absolute and relative lymphocyte counts than PCR+Pn− patients (respectively: 1.60 ± 0.62 * 10^9^/L vs. 2.22 ± 0.75 * 10^9^/L, p = 0.0222; 22.74 ± 8.47% vs. 33.06 ± 10.50%, p = 0.0047).

As regards monocyte (MONO) count, PCR−Pn+ patients showed a significant increase of their absolute count compared to all other groups, but such a difference was largely reduced with the percentage count for which the statistical significance was reached only with respect to the control group.

The acute inflammatory parameters clearly reflected the presence/absence of pneumonia: indeed, both PCR−Pn+ and PCR+Pn+ groups showed a significant increase of both ESR (compared to controls) and CRP (compared to both controls and PCR+Pn− patients). Notably, neither ESR nor CRP were significantly different between PCR−Pn+ and PCR+Pn+ groups.

### General lymphocyte immunophenotyping

The general lymphocyte subpopulations are also described in Table 3. As regards B cells (CD19+CD3−), all three respiratory disease groups (PCR−Pn+, PCR+Pn+, and PCR+Pn−) showed a relative and absolute increase of these lymphocytes; however, such an increase was greater and reached a significant difference with control groups only in PCR−Pn+ group (0.325 ± 0.329 * 10^9^/L vs. 0.183 ± 0.090 * 10^9^/L, p = 0.0001; 15.86 ± 9.02% vs. 10.42 ± 4.39%, p = 0.0110). Interestingly, the absolute B cell count also showed a mildly significant difference between PCR−Pn+ and PCR+Pn+ patients (p = 0.0107). No clear trend was observed for NK cells (CD3−CD56+) compared to controls, even though both pneumonia groups (PCR−Pn+ and PCR+Pn+) showed a lower absolute count compared to PCR+Pn− patients (respectively: 0.218 ± 0.184 * 10^9^/L and 0.217 ± 0.130 * 10^9^/L, vs. 0.385 ± 0.271 * 10^9^/L; respectively, p = 0.0031 and p = 0.0094). Overall, no evident differences among these four groups were observed as regards T cells (CD3+CD19−CD56−), including their main subpopulations: CD4+T, CD8+ T, DNT (CD8−CD4−), and DPT (CD4+CD8+) cells. Actually, a mildly significant difference was observed in the relative and absolute count of DNT cells, which were increased only in PCR+Pn− patients compared to both pneumonia groups (PCR−Pn+ and PCR+Pn+). Notably, the relative and absolute counts showed the same trend for all these lymphocyte subpopulations, overall.

### Memory T cells analysis

**Fig. 2:**
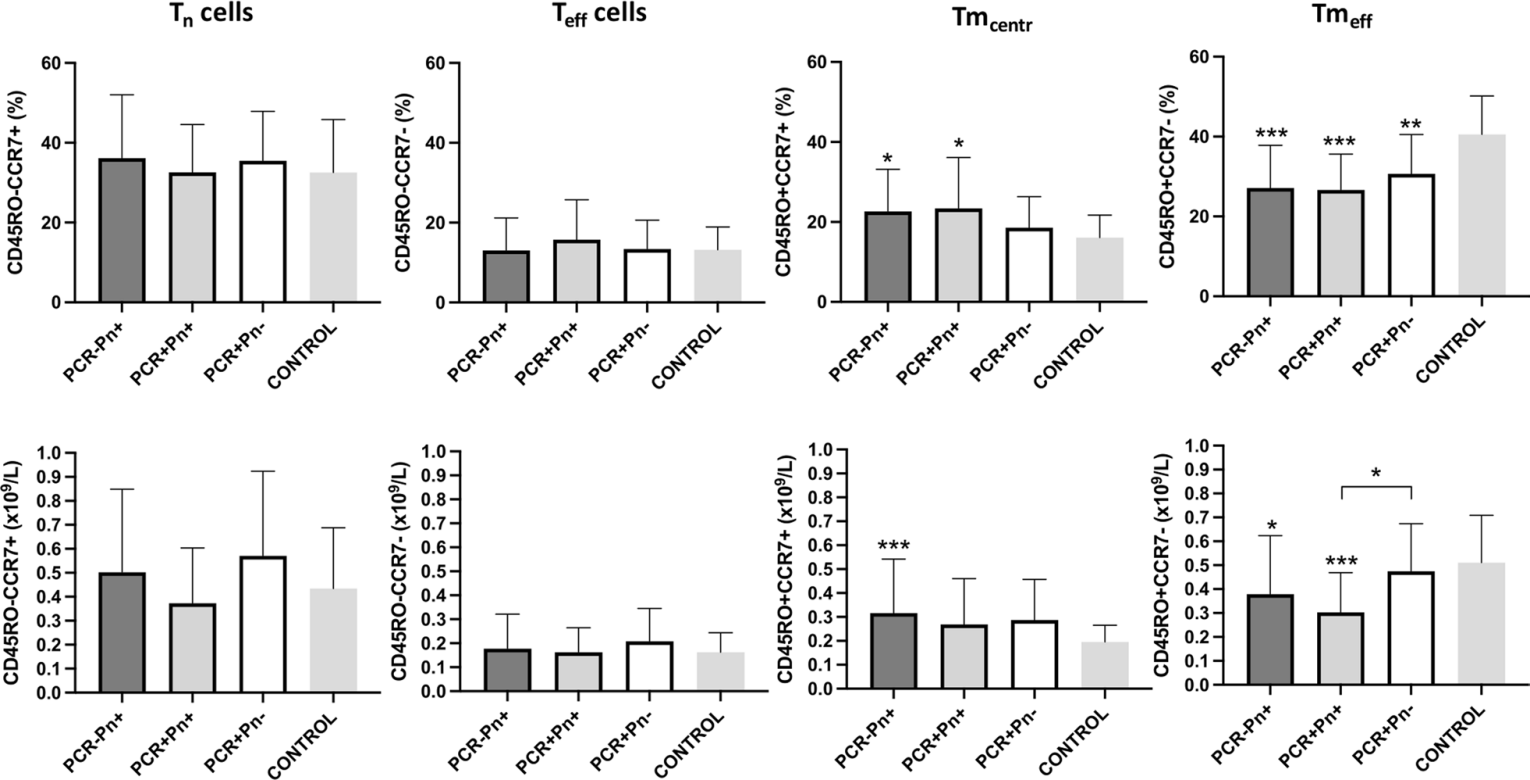
Naïve (T_n_), effector (T_eff_), central memory (Tm_centr_) and effector memory (Tm_eff_) T cells subsets in the four study groups (upper row: percentage count; lower row: absolute count)

First, we analyzed the expression of the main immunological markers defining the naïve, effector and memory phenotype of T cells, namely CD45RO and CCR7. Among T lymphocytes, naïve T cells (T_n_; CD45RO−CCR7+), effector T cells (T_eff_; CD45RO−CCR7−), memory central T cells (Tm_centr_; CD45RO+CCR7+) and memory effector T cells (Tm_eff_; CD45RO+CCR7−) have been measured, as shown in Fig. 2.

A clear unbalance in the T memory compartment emerged from this analysis on the whole pool of T (CD3+) cells, showing a significant percentage increase in Tm_centr_ cells (PCR−Pn+: 22.61 ± 10.55%, p = 0.0184; PCR+Pn+: 23.38 ± 12.75%, p = 0.0224) and, conversely, a significant percentage decrease in Tm_eff_ cells (PCR−Pn+: 27.11 ± 10.73%, p < 0.0001; PCR+Pn+: 26.62 ± 9.01%, p < 0.0001) in both pneumonia groups compared to the controls (Tm_centr_ cells: 16.05 ± 5.69%; Tm_eff_ cells: 40.51 ± 9.67%). It is interesting to notice that PCR+Pn− group (Tm_centr_ cells: 18.58 ± 7.74%; Tm_eff_ cells: 30.67 ± 9.83%) ranked between patients with pneumonia (PCR−Pn+ and PCR+Pn+) from one side and the control group from the other side: however, the differences between PCR+Pn− patients and all the other groups did not reach any statistical significance, except for Tm_eff_ cells versus the control group (p = 0.0070).

Compared to the control group (Tm_centr_ cells: 0.195 ± 0.070 * 10^9^/L; Tm_eff_ cells: 0.511 ± 0.198 * 10^9^/L), the trend described above in the memory T compartment for the PCR−Pn+ (Tm_centr_ cells: 0.316 ± 0.226 * 10^9^/L, p = 0.0001; Tm_eff_ cells: 0.379 ± 0.244 * 10^9^/L, p = 0.0293), PCR+Pn+ (Tm_centr_ cells: 0.269 ± 0.191 * 10^9^/L, p = ns; Tm_eff_ cells: 0.303 ± 0.166 * 10^9^/L, p = 0.0009), and PCR+Pn− (Tm_centr_ cells: 0.287 ± 0.170 * 10^9^/L, p = ns; Tm_eff_ cells: 0.474 ± 0.200 * 10^9^/L, p = ns) groups, was also maintained by expressing lymphocyte subpopulation as absolute numbers. Notably, the gap in Tm_eff_ cells between PCR+Pn+ and PCR+Pn− groups was accentuated when these cells are expressed in absolute terms (respectively: 0.303 ± 0.166 * 10^9^/L vs. 0.474 ± 0.200 * 10^9^/L, p = 0.0274).

**Fig. 3:**
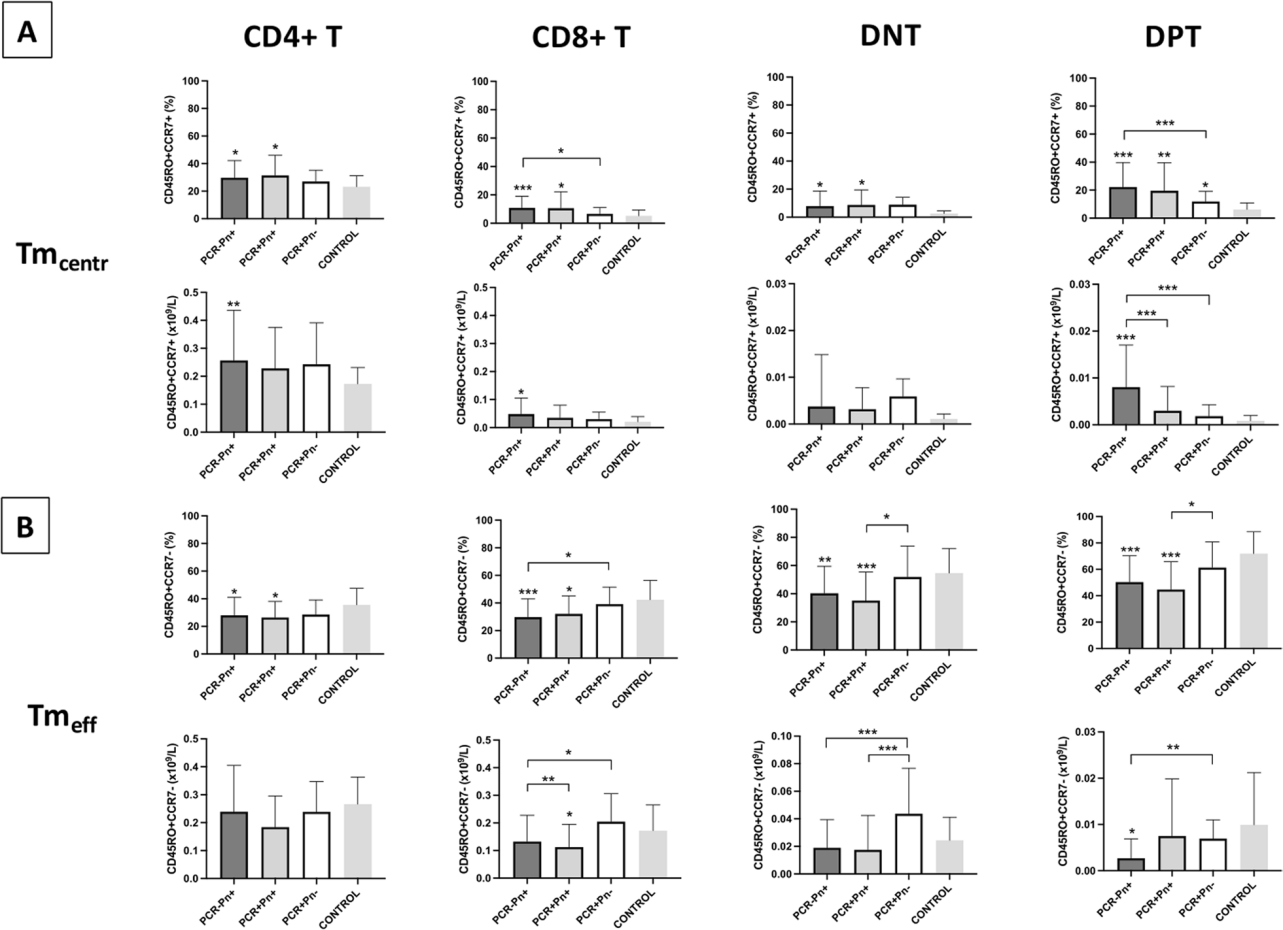
Analysis of central (**A**) and effector (**B**) memory T cells in the main T-cell populations based on CD4/CD8 expression in the four study groups (upper row: percentage count; lower row: absolute count)

We also analyzed this perturbation in the memory T cell compartment inside each one of the four T cell subpopulations identified by the differential expression of CD4 and/or CD8 markers. As shown in Fig. 3, the increase of Tm_centr_ cells (panel A, upper row) and the reduction of Tm_eff_ cells (panel B, upper row) was maintained in all these T cell subpopulations (CD4+, CD8+, DNT, and DPT) in percentage terms. Overall, a gradual trend from controls to PCR+Pn+ patients, passing through intermediate values of PCR+Pn− groups was observed in each of these T subpopulations individually. In general, PCR−Pn+ patients showed values comparable to PCR+Pn+ group. We also expressed all these cell populations as absolute count, and this trend characterized by higher Tm_centr_ cells (panel A, lower row) and lower Tm_eff_ (panel B, lower row) was still present, overall. The corresponding numerical data are shown in Table 4.

**Table 4:** Analysis of central and effector memory T cells in the main T-cell populations based on CD4/CD8 expression in the four study groups

	PCR−Pn+	PCR+Pn+	PCR+Pn−	Controls	p-value
CD4+ T cells					
CD45RO+CCR7+ (% CD4+T)	29.59 ± 12.56	31.40 ± 14.62	27.00 ± 8.25	23.25 ± 7.99	0.0077
* CD45RO*+*CCR7*+ *(10*^*9*^*/L)*	0.2563 ± 0.1799	0.2281 ± 0.1468	0.2427 ± 0.1481	0.1727 ± 0.0583	0.0014
CD45RO+CCR7− (% CD4 + T)	27.94 ± 13.14	26.40 ± 11.68	28.57 ± 10.53	35.55 ± 11.96	0.0191
* CD45RO*+*CCR7− (10*^*9*^*/L)*	0.2388 ± 0.1661	0.1838 ± 0.1116	0.2385 ± 0.1087	0.2661 ± 0.0969	ns
CD8+T cells					
CD45RO+CCR7− (% CD4+T)	10.80 ± 8.20	10.57 ± 11.55	6.57 ± 4.50	5.11 ± 4.14	< 0.0001
* CD45RO*+*CCR7*+ *(10*^*9*^*/L)*	0.0484 ± 0.0575	0.0347 ± 0.0450	0.0307 ± 0.0248	0.0207 ± 0.0188	0.0406
CD45RO+CCR7− (% CD8+T)	29.71 ± 13.22	32.02 ± 13.10	39.15 ± 12.36	42.33 ± 14.03	< 0.0001
* CD45RO*+*CCR7− (10*^*9*^*/L)*	0.1323 ± 0.0955	0.1122 ± 0,0826	0.2044 ± 0,1017	0.1718 ± 0.0933	0.0013
DNT cells					
CD45RO+CCR7+ (% DNT)	7.89 ± 10.77	8.75 ± 10.72	8.90 ± 5.29	2.54 ± 1.97	0.0327
* CD45RO*+*CCR7*+ *(10*^*9*^*/L)*	0.0037 ± 0.0110	0.0032 ± 0.0046	0.0059 ± 0.0038	0.0011 ± 0.0010	ns
CD45RO+CCR7− (% DNT)	40.31 ± 19.18	35.14 ± 20.28	51.86 ± 22.02	54.66 ± 17.45	0.0001
* CD45RO*+*CCR7− (10*^*9*^*/L)*	0.189 ± 0.0205	0.174 ± 0.0249	0.0437 ± 0.0331	0.243 ± 0.0168	0.0148
DPT cells					
CD45RO+CCR7+ (% DPT)	22.17 ± 17.55	19.56 ± 10.07	11.84 ± 7.28	6.22 ± 4.65	< 0.0001
* CD45RO*+*CCR7*+ *(10*^*9*^*/L)*	0.0080 ± 0.0089	0.0030 ± 0.0052	0.0018 ± 0.0025	0.0008 ± 0.0011	< 0.0001
CD45RO+CCR7− (% DPT)	50.31 ± 19.98	44.58 ± 21.22	61.31 ± 19.46	71.85 ± 16.61	< 0.0001
* CD45RO*+*CCR7− (10*^*9*^*/L)*	0.0027 ± 0.0042	0.0075 ± 0.0124	0.0069 ± 0.0040	0.0099 ± 0.0113	0.001

With respect to COVID-19 patients, some interesting differences are displayed by the unconventional DNT and DPT populations, especially if expressed as percentages. In particular, as regards Tm_eff_ characterized by CD4−CD8− (DNT) immunophenotype, whatever the cell count was expressed (absolute number or percentage of the whole DNT population), PCR+Pn+ and PCR+Pn− groups were significantly different (respectively: 35.14 ± 20.28% vs. 51.86 ± 22.02%, p = 0.0133; 0.0174 ± 0.0249 * 10^9^/L vs. 0.0437 ± 0.0331 * 10^9^/L, p = 0.0003). PCR−Pn+ patients (0.0189 ± 0.0205 * 10^9^/L) showed absolute values similar to PCR+Pn+ patients, but both groups did not significantly differ from the controls (0.0243 ± 0.0168 * 10^9^/L), unlike the PCR+Pn− group (p = 0.0239), which was also characterized by an increased number of total DNT cells compared to all other three groups, as previously described. Indeed, if DNTm_eff_ were expressed in relative terms (as percentage of DNT), both PCR−Pn+ and PCR+Pn+ groups (respectively: 40.31 ± 19.18%, p = 0.0046; 35.14 ± 20.28%, p = 0.0005) were significantly different from the controls (54.66 ± 17.45%).

## Discussion

Central and effector memory T (Tm_centr_ and Tm_eff_) cells represent the two major subsets of the memory T-cell pool. Both are characterized by the expression of the CD45RO isoform and, conversely, by the lack of expression of the CD45RA isoform (CD45RO+CD45RA−), but they differ for the expression of the lymph node-homing CC‑chemokine receptor 7 (CCR7): accordingly, CD45RO+CCR7+Tm_centr_ cells traffic to lymphoid tissues, and CD45RO+CCR7−Tm_eff_ cells can migrate to multiple peripheral tissue sites [12, 13].

In this preliminary study, the main and novel finding is that patients with ongoing and acute respiratory infections leading to interstitial pneumonia developed an unbalance inside these two main T-memory populations; notably, these alterations were observed in both SARS-CoV-2 positive and negative patients developing interstitial pneumonia. In detail, Tm_centr_ cells increased, whereas Tm_eff_ cells decreased in PCR−Pn+ and PCR+Pn+ patients in relative (percentage) and absolute terms. Overall, this unbalance is similarly present in all the four T cell subpopulations based on the differential expression of CD4 and CD8 markers, namely CD4+, CD8+, DNT and DPT cells.

Focusing on COVID-19 patients, we should first underline that there is a relatively greater number of clinical studies investigating SARS-CoV-2 antigen-specific T cell subsets (including memory T cells) in the convalescent and, to a lesser extent, acute COVID-19 patients [14–18], compared to the number of papers assessing the general (non-antigen specific) memory T cell populations in the peripheral blood, especially during the acute phase of COVID-19, which represents the main target of the present research.

As an additional premise useful to better discuss and consider our results on memory T cells homeostasis in peripheral blood, it should be highlighted that, in terms of general lymphocyte subpopulations (CD4/CD8+/− T, B and NK cells), there are numerous studies: most of them suggested that all main lymphocyte subsets tend to be below the normal ranges during the acute and early phase of COVID-19 [7]. Several studies (almost all of which carried out in 2020, thus during the first pandemic wave) suggested that such alterations of the general lymphocyte subpopulations could correlate with disease severity and outcome [7, 19–21]. For instance, Deng et al*.* described that, at 1 week after the onset of illness, CD3+, CD4+, and CD8+ counts were significantly lower in patients with severe COVID-19 compared to those with non-severe disease [22]. However, this analysis was probably performed after starting the COVID-19 therapy (including steroid and intravenous immunoglobulin), which may have affected the peripheral lymphocyte homeostasis to some extent. In our present study, lymphocyte immunophenotyping was done at the hospital admission and we observed no general alteration of CD3+, CD4+ and CD8+ cells in COVID-19 patients compared to both controls and SARS-CoV-2 PCR negative patients with pneumonia. No clear unbalance was evident in B-cell and NK-cell compartments of COVID-19 patients. We only observed mild differences inside COVID-19 patients, between those with and without pneumonia: PCR+Pn− patients showed an increase in the relative and absolute number of NK and DNT cells compared to both controls and PCR+Pn+ patients. Of course, we cannot certainly explain our different observations, but a lower rate of clinically severe COVID-19 patients in our study population (despite the concomitant diagnosis of interstitial pneumonia) may have contributed; indeed, no deaths were recorded among our COVID-19 patients in the acute phase.

Coming back to our main findings, namely the general increase in central T-memory cells and decrease in effector T-memory cells in both PCR+Pn+ and PCR−Pn+ patients, this is the first time that such a specific aspect has been clearly highlighted and, actually, linked to the occurrence of interstitial pneumonia rather than to a specific infectious disease, such as COVID-19. Indeed, PCR−Pn+ groups included patients who were not only SARS-CoV-2 PCR negative, but also negative for IgM specific to SARS-CoV-2. Therefore, we could suppose that they were likely to be affected by a different acute viral infection, according to serological and molecular guidelines for COVID-19 diagnosis [6].

There are few studies including general Tm_centr_ and Tm_eff_ cells analysis. Odak et al. compared severe and mild COVID-19 with a control group. In this regard, their results were not similar to ours. Very briefly, in terms of Tm_eff_ cells they observed a decrease in the CD4+ T-cell pool, and an increase in the CD8+ T-cell pool (but only in severe patients), compared to controls. As regards Tm_centr_ cells, they found an increase in the CD4+ T-cell population only in mild patients, and a decrease in the CD8+ T-cell pool only in severe patients, compared to controls. However, a large heterogeneity of samples collection timing (3–32 days after the onset of symptoms; > 75% of samples collected after 8 days) characterized this study [23]. Rajamanickam et al*.* described the longitudinal dynamics of circulating T cell subsets in 46 COVID-19 patients starting from 15–30 days until 180 days from the diagnosis. In terms of Tm_centr_ and Tm_eff_ cells, during this follow-up they described a progressive increase of both memory types for CD4+ compartment, whereas no significant changes were described in CD8+ T memory cellular pool. However, these values were not compared to any control group, and this study may have missed the earliest phase of COVID-19 clinical course [24]. Another research by Kalpacki et al*.* assessed the general T memory compartment in a similar way as our approach. They compared clinically severe (n = 20) and non-severe (n = 20) COVID-19 patients and reported a general reduction of Tm_centr_ and Tm_eff_ cells pool, especially as regards CD4+ T cells. Unfortunately, they did not provide any information on the radiological aspect (and, thus, lung involvement) and did not include any control group, even though they also assessed central and effector memory T cells at the disease onset [25]. Very recently, De Biasi et al*.* also included a memory T-cell analysis in 39 patients with SARS-CoV-2 pneumonia: they showed “no gross changes between patients and controls […] simply using markers related to naïve, memory or effector cells” [26]. Conversely, our results showed some similarities with a recent study by Adamo et al.: these authors also observed a clear percentage increase of CD4+ Tm_centr_ cells, which was more accentuated in severe patients than in mild ones, whereas a similar increase for CD8+ Tm_centr_ cells was observed in severe group only; moreover, they also reported a decrease in CD4+ Tm_eff_ cells, but not in CD8+ Tm_eff_ cells, except for severe cases. Notably, the same analysis expressed as absolute counts provided similar trends for Tm_eff_ cells overall, but opposite trends Tm_centr_ cells, in both CD4+ and CD8+ T-cell compartments [27].

Therefore, our study can add further data regarding the general assessment of Tm_centr_ and Tm_eff_ cells in COVID-19 patients. Indeed, as mentioned, most clinical studies investigating general homeostasis of circulating lymphocytes, did not assess specific memory T cells markers [7, 28, 29]. Accordingly, Lagadinou et al. recently reviewed the alterations of lymphocyte subsets SARS-CoV-2 pneumonia, but they reported no analysis on memory T cells [30]. Moreover, we think it is important to further highlight that, in our study, the increase in Tm_centr_ cells and decrease in and Tm_eff_ cells affected all four main CD4/CD8 subtypes T cells (CD4 + T, CD8 + T, DNT, and DPT cells) and was similar in both SARS-CoV-2 PCR+ and SARS-CoV-2 PCR− pneumonia groups (PCR+Pn+ and PCR−Pn+, respectively). Notably, SARS-CoV-2 PCR+ patients without pneumonia (PCR+Pn−) in general ranked between patients with pneumonia (PCR+Pn+ and PCR−Pn+) from one side and controls from the other side, as regards both Tm_centr_ and Tm_eff_ cells. This observation might suggest a kind of trend related to the “depth” of the respiratory disease, according to the presence of lung involvement. In other words, we may speculate that, in the setting of respiratory diseases, the general perturbation of Tm_centr_ and Tm_eff_ cells, as observed in our study, may be more related to the presence or absence of pneumonia rather than the specific etiology, namely SARS-CoV-2 or other etiologic agents (presumably, viral) in SARS-CoV-2 negative cases.

Even though memory T cells subsets specific to SARS-CoV-2 antigens can be currently detected, this analysis is not available to clinical laboratories and requires advanced research facilities [31]. Therefore, if our findings could be confirmed by further and independent studies, our relatively simple flow cytometry panel may be within the reach of the clinical laboratory: thus, the general assessment of Tm_centr_ and Tm_eff_ cells balance may be further investigated as a potential biological marker of lung involvement (and/or disease severity) in patients affected with viral respiratory infections, including COVID-19.

Several limitations affected our present study. The samples of SARS-CoV-2 PCR positive patients were relatively small, especially as regards the sub-analysis between patients with and without pneumonia: indeed, the study period did not fall into an epidemic peak of SARS-CoV-2 infection in Kazakhstan. Moreover, the SARS-CoV-2 variants affecting our patients are not known. In this regard, it is important to notice that most (if not all) the previous studies on these main lymphocyte subsets were carried out in 2020, whereas our study period completely falls into 2022: therefore, we can also assume that our patients were affected with different SARS-CoV-2 variants from patients included in those previous studies, which should be also taken in account as an additional and potential explanation for different findings among all these studies. Finally, no longitudinal data are currently available in order to describe the dynamics of memory T cells perturbation described in the present study.

## Conclusion

Overall, we observed both absolute and relative increases of Tm_centr_ cells and decrease of Tm_eff_ cells in patients affected with interstitial pneumonia (regardless of the positive or negative results of SARS-CoV-2 PCR), compared to controls. This trend was also noticed for SARS-CoV-2 PCR positive patients without pneumonia, even though it was less accentuated and not significant. These findings need additional research to be confirmed and should be completed by longitudinal studies, in order to consider this relatively simple lymphocyte immunophenotyping as a potential biological marker of lung involvement (and/or disease severity) in patients affected with viral respiratory infections.

## Data Availability

Data cannot be released without patients’ consent in the public domain for open, unrestricted access. Researchers who are interested and meet the criteria for research access to our data may refer to the corresponding author.
